# Caregivers with limited English proficiency: Satisfaction with primary pediatric healthcare

**DOI:** 10.1177/13674935241252479

**Published:** 2024-05-08

**Authors:** Linda Thanh Duong, My-An Tran

**Affiliations:** 1 Faculty of Health Sciences, 3710 McMaster University, Hamilton, ON, Canada; 2 Pediatric Neurology, 27338 Children’s Hospital of Eastern Ontario, Ottawa, ON, Canada

**Keywords:** Communication, health status disparities, language, patient satisfaction, physician–patient relations

## Abstract

With a growing 25.5 million people in the United States experiencing limited-English proficiency (LEP), there is a concern over these individuals’ experiences in healthcare. Health outcomes of LEP status are well-documented for adults in hospitals; however, less is known about patient experience, pediatric populations, and primary care settings. This study investigated differences in caregiver satisfaction between families with and without LEP receiving healthcare for their child. A sample of 25,118 caregivers whose children from birth to 17 years had met with any healthcare providers in the past year was used. Analyses consisted of unpaired *t*-tests comparing mean satisfaction of LEP and English-proficient (EP) caregivers in the domains of how often primary healthcare providers *spent enough time with the child*, *listened*, *provided specific information*, *demonstrated sensitivity to the family’s values*, and *made the respondent feel like a partner*. In all aspects of caregiver satisfaction, mean satisfaction scores were significantly lower for LEP caregivers than EP caregivers. The largest drops were seen in perceived time and sensitivity. These results highlight a need to ensure LEP families receive equitable and high-quality primary care services, ultimately building trust in the healthcare system and improving children’s health and well-being.

## Introduction

In the United States, there are an estimated over 25.5 million people who experience limited-English proficiency (LEP) (Proctor et al., 2018). These individuals may represent Indigenous communities, newcomers, and people who are deaf or hard-of-hearing (Bowen, 2001). With constant shifts in migration, the United States’ linguistic diversity is expected to continue increasing in future years (Yeheskel and Shail, 2018) particularly for those of working age with families or looking to start a family (Organisation for Economic Co-operation and Development, 2017: 107–166). In most countries with large LEP populations, designing interventions that promote integration into everyday life is standard; children and their families learn to hold conversations, visit public spaces, and attend school or work (Hall et al., 2021). Few programs, however, have aimed to develop these individuals’ health literacy, which consists of nuanced and precise medical vocabulary (Liu et al., 2020). Healthcare interactions involving both LEP and a lack of health literacy can be challenging (Hall et al., 2021).

For general adult populations, impacts of LEP status compared to English proficiency (EP) status are somewhat well-documented, especially in North American emergency departments. Patients facing LEP can expect a longer length of stay (John-Baptiste et al., 2004), an increased likelihood of adverse events (Dobson, 2007), and higher risks of readmission (Karliner et al., 2017) than patients without LEP. Taken together, lacking infrastructure to work with LEP status impacts patient care. In addition to this, as most providers see patients with LEP daily or weekly, lacking LEP adaptation strategies places great financial and resourceful strain on the healthcare system (Hasnain-Wynia et al., 2006).

A typical solution to working with LEP-status patients is to receive translation through a family member, friend, or multi-lingual staff member; however, without confidence in the ad hoc interpreter’s proficiency with medical terminology and ethical training, information is often modified, added, or omitted (Goldman, 2017). Inaccurate communication places patients under an increased risk of harm (Goldman, 2017). A major alternative is acquiring medically trained interpretive services to alleviate cultural and linguistic barriers (Herzberg et al., 2021). Although promising in theory, the United States lacks federal regulations and guidance with regards to using certified interpreters (Jacobs et al., 2018). Most medical centers also cite cost as a barrier to access (Jacobs et al., 2018). Due to this lack of standardization and accessibility, various physician and patient sources have pointed out conflicting results while using interpretation (Flores, 2005).

Issues surrounding adverse events, misinterpretation, and lack of regulation prompt questioning into how LEP status fares in non-urgent/non-intensive settings and different patient populations. In 2016, children from birth to 17 years visited primary care physicians more often than any other age group (Ashman et al., 2019). In the United States, primary care refers to an integrated, broad range of healthcare services involving professionals who form long-term partnerships with families and communities (Donaldson et al., 1996). These partnerships prevent disease and promote health, guiding patients through health decisions and resources (Donaldson et al., 1996). Nearly all Americans receive some form of primary care (Levine et al., 2019) within family medicine, general internal medicine, or general pediatrics (Phillips, 2005). Little is known about how LEP status impacts children in primary care (Karliner et al., 2017). In addition, a scan through past literature finds that most studies have focused on patient outcomes and safety with less regard to patient experience. Interacting with infants, children, and adolescents from LEP households requires specific attention. Unlike caring for independent adults, pediatric primary care providers must also consider the information, comfort, advocacy, and consent that a caregiver can provide for their child (Coughlin, 2018). Further, a family’s satisfaction can increase their trust in the service (Flores, 2005). Trust leads to more frequent and thorough assessments, earlier intervention and, ultimately, better child and lifetime health outcomes (Flores, 2005). With this, it is important to determine whether LEP status in pediatric primary care exacerbates existing challenges with patient experience and provider–patient communication.

## Aim

The aim was to compare LEP to EP caregiver satisfaction with pediatric primary healthcare and to identify aspects of satisfaction with the largest disparities.

## Methods

### Procedures

This study’s sample was drawn from the National Survey of Children’s Health (NSCH), an annual survey conducted in the United States, to generate data on physical and mental health of children from birth to 17 years. This present study uses data from the NSCH 2019 sample. Although more recent data from 2020 is available, COVID-19-related service disruptions and introduction of remote care would likely hinder this paper’s findings and relevance. Ethics for the NSCH were not sent to an institutional review board but rather approved by the United States Census Bureau and Office of Management and Budget as it was considered exempt research under Title 45, Part 46 from the Code of Federal Regulations. As this is a source of secondary data, approval to conduct analyses and write this paper from local review boards was not required.

The United States Census Master Address File was used to identify 180,000 addresses where children under 18 resided or were likely to reside. A stratified sampling strategy was used. Addresses directly linked to children were oversampled, whereas addressed with a high probability of child presence were moderately sampled; addresses with low probabilities of child presence were excluded from sampling. Between June 2019 and January 2020, consenting children’s primary caregiver completed (1) a screener questionnaire to determine their eligibility for the study and (2) a topical questionnaire to collect their children’s health data via phone, paper, or web instrument. In total, both questionnaires took approximately 37 min to complete. Questionnaires were available in English and Spanish for all methods of collection with additional languages available via telephone. Nonresponse bias analyses conducted by the NSCH found no significant systematic biases in their sampling strategy.

For this study, caregivers with a child below the age of 18 who responded “yes” to seeing any healthcare provider in the past 12 months were included.

### Measures

*English Proficiency* was determined through respondents’ household languages. Participants were asked the primary language spoken at home with options to select between “English,” “Spanish,” or “other.” Those who spoke English at home are henceforth referred to as the EP sample and those who spoke Spanish or another language are henceforth referred to as the LEP sample.

*Caregiver Satisfaction* was measured using five items that assessed how often healthcare providers (1) *spent enough time with the child*, (2) *listened carefully*, (3) *provided specific information*, (4) *demonstrated sensitivity to the family’s values and customs*, and (5) *made the respondent feel like a partner in their child’s care*. Each item was measured on a four-point scale (1 = *Never*, 4 = *Always*). Items were adapted from a scale intended to assess characteristics of primary pediatric care developed by The Child and Adolescent Health Measurement Initiative (CAHMI) with recommendations from the American Academy of Pediatrics (CAMHI, 2009). These domains were selected for their ability to subjectively measure whether a family’s expectations regarding healthcare encounters were met. Although a definition for patient satisfaction has long been debated (Gill, 2009), these outcomes reflect general themes of communication, respect, and cooperation agreed upon by most authors. Cronbach’s alphas test measured reliability of the service quality scale which yielded internal consistency with alphas 0.903 and 0.920 for the LEP and EP samples, respectively.

*Demographic information* consisted of sex, age, and race. Sex was coded as male or female. Age (in years) was calculated using birth year; January 1 was assumed to be the specific birth date. Race was coded as white only, Black/African American only, or other.

### Data analysis

Statistical analyses were conducted using R (https://www.r-project.org/) and R Studio Version 4.2.0. Descriptive statistics were performed. The Kolmogorov–Smirnov test was used to assess normality. Pearson’s correlations were then completed to investigate strengths of association between caregiver satisfaction variables. To compare satisfaction across samples, non-parametric and parametric tests were run. As outcomes were ordinal in nature and were not normally distributed, the non-parametric Mann–Whitney *U* test was first used to compare LEP rank sums to EP rank sums for all aspects of caregiver satisfaction, with the rank-biserial correlation used for effect size (very low = 0 < 0.2, low = 0.2 < 0.4, moderate = 0.4 < 0.6, strong = 0.6 < 0.8, and very strong = 0.8 < 1) (Bartz, 1999). Following this, independent sample *t*-tests were performed to test differences in LEP mean and EP mean in each satisfaction item. Cohen’s *d* was used to determine effect size (small = 0.2, medium = 0.5, and large = 0.8) (Cohen, 1988). As results from both tests were comparable, findings from parametric tests are presented for ease of interpretation and visualization. Further information on results of non-parametric testing is included in the Appendix. Given the large sample available for calculations, statistical significance was considered when *p* < .001.

## Results

In 2019, 29,433 (42.4%) households responded to the NSCH topical questionnaire. Most respondents (25,118; 85.3%) answered “yes” to seeing any healthcare provider in the last year.

The LEP sample was smaller than the EP sample, which was expected as individuals with LEP compare to EP at a ratio of approximately 1 to 10 nationally. Mean ages and proportion of sex were relatively uniform across samples. In the LEP sample, 699 (49.2% of LEP respondents) spoke Spanish, whereas 722 (50.8%) spoke another, unspecified language. Additionally, the LEP sample also saw higher proportions of households identifying their child as a racialized individual (i.e., not identifying as “White only”). Sample characteristics are summarized in Table 1.

**Table 1.** Demographic characteristics of children from households with limited English proficiency or with English proficiency.

Characteristic	LEP sample (*n* = 1421)	EP sample (*n* = 23,697)
Age		
Mean age (*SD*), years	8.9 (5.3)	9.7 (5.2)
Sex		
Male *n* (%)	754 (53.1)	12320 (52.0)
Female *n* (%)	667 (46.9)	11377 (48.0)
Race		
White only *n* (%)	826 (58.1)	19162 (80.9)
Black/African American only *n* (%)	84 (5.9)	1592 (6.7)
Other *n* (%)	511 (36.0)	2943 (12.4)
Household language		
Spanish *n* (%)	699 (49.2)	
Other *n* (%)	722 (50.8)	

*n*: sample size; *SD*: standard deviation.

Satisfaction items were positively, moderately to strongly correlated with one another in both samples. This is evidence that measures used in this study have convergent validity in assessing caregiver satisfaction. Results of Pearson’s correlation matrix are summarized in Tables 2 and 3.

**Table 2.** Strength of association between aspects of caregiver satisfaction in limited English proficiency sample.

In the past 12 months, how often did this child’s doctors or other healthcare providers		Spend enough time with child	Listen to respondent	Provide specific information about child	Show sensitivity to family’s values and customs	Help respondent feel like a partner in child’s care
Spend enough time with child	*r* [99% CI]	1.00 [1.00, 1.00]				
Listen to respondent	*r* [99% CI]	0.63 [0.59, 0.67]	1.00 [1.00, 1.00]			
Provide specific information about child	*r* [99% CI]	0.58 [0.53, 0.62]	0.70 [0.66, 0.73]	1.00 [1.00, 1.00]		
Show sensitivity to family’s values and customs	*r* [99% CI]	0.60 [0.55, 0.64]	0.72 [0.69, 0.75]	0.70 [0.66, 0.73]	1.00 [1.00, 1.00]	
Help respondent feel like a partner in child’s care	*r* [99% CI]	0.60 [0.55, 0.64]	0.68 [0.64, 0.72]	0.72 [0.69, 0.75]	0.69 [0.65, 0.72]	1.00 [1.00, 1.00]

Analyses aimed to find correlations between various aspects of caregiver satisfaction.

*r*: Pearson correlation coefficient; 99% CI: 99% confidence interval.

**Table 3.** Strength of association between aspects of caregiver satisfaction in English proficiency sample.

In the past 12 months, how often did this child’s doctors or other healthcare providers		Spend enough time with child	Listen to respondent	Provide specific information about child	Show sensitivity to family’s values and customs	Help respondent feel like a partner in child’s care
Spend enough time with child	*r* [99% CI]	1.00 [1.00, 1.00]				
Listen to respondent	*r* [99% CI]	0.71 [0.7, 0.72]	1.00 [1.00, 1.00]			
Provide specific information about child	*r* [99% CI]	0.64 [0.63, 0.65]	0.74 [0.73, 0.75]	1.00 [1.00, 1.00]		
Show sensitivity to family’s values and customs	*r* [99% CI]	0.62 [0.61, 0.63]	0.74 [0.73, 0.75]	0.73 [0.72, 0.74]	1.00 [1.00, 1.00]	
Help respondent feel like a partner in child’s care	*r* [99% CI]	0.64 [0.63, 0.65]	0.74 [0.73, 0.75]	0.76 [0.75, 0.77]	0.72 [0.71, 0.73]	1.00 [1.00, 1.00]

Analyses aimed to find correlations between various aspects of caregiver satisfaction.

*r*: Pearson correlation coefficient; 99% CI: 99% confidence interval.

**Figure 1.**
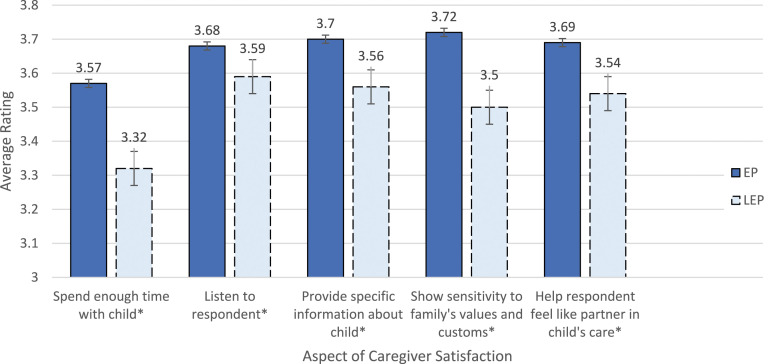
Difference in average caregiver satisfaction between families with limited English proficiency and with English proficiency. *LEP*: *limited English proficiency sample; EP*: *English proficiency sample;* *, statistically significant (*p* < .001). 99% confidence intervals are shown. Analyses aimed to find differences in mean scores of caregiver satisfaction.

All five aspects of caregiver satisfaction were, on average, adequate for both LEP and EP families. The average responses fell between “*Sometimes*” and “*Always*.” However, in all regards, LEP satisfaction mean (*M*_LEP_[SD]) was lower than EP satisfaction mean (*M*_EP_[SD). Table 4 and Figure 1 summarized means and results from the unpaired *t*-tests. There was a difference (−0.25, 99% CI [–0.31, −0.19]) in perceived time healthcare providers spent with LEP families (*M*_LEP_ = 3.32 (0.83)) compared to EP families (*M*_EP_ = 3.57 (0.54)), (*d* = 0.38; *t* (1,524) = −11.15). This was the biggest observed difference and second largest effect size. Likewise, results demonstrated that LEP families (*M*_LEP_ = 3.59 (0.63)) felt they were carefully listened to less frequently (−0.09, 99% CI [–0.14, −0.05]) than EP families (*M*_EP_ = 3.68 (0.56)), (*d* = 0.16; *t* (1,558) = −5.36), while receiving less specific information (−0.14, 99% CI [–0.20, −0.10]) about their child (*M*_LEP_ = 3.56 (0.69); *M*_EP_ = 3.70 (0.55)), (*d* = 0.26; *t* (1531.3) = −7.91). LEP families (*M*_LEP_ = 3.50 (0.74)) also saw decreased (−0.22, 99% CI [–0.27, −0.17]) perceived sensitivity to their values and customs (*M*_EP_ = 3.71 (0.55)), (*d* = 0.39; *t* (1,515) = −10.98). This was the second largest difference and biggest effect size. Finally, LEP families (*M*_LEP_ = 3.54 (0.71)) felt less frequently (−0.15, 99% CI [–0.20, −0.10]) that they were a partner in their child’s care (*M*_EP_ = 3.69 (0.58)), (*d* = 0.26; *t* (1,537) = −8.04).

**Table 4.** Results of unpaired *t*-tests comparing caregiver satisfaction with provider between families with limited English proficiency and with English proficiency.

		*M (SD)*	*M* difference [99% CI]	*t*	Df	Sig (2-tailed)	*d* [99% CI]
Spend enough time with child	EP	3.57 (0.65)	−0.25 [−0.31, −0.19]	−11.15	1524	2.20 × 10^−16^	0.38 [0.33, 0.43]
	LEP	3.32 (0.83)					
Listen to respondent	EP	3.68 (0.56)	−0.09 [−0.14, −0.05]	−5.36	1558	9.73 × 10^−8^	0.16 [0.10, 0.23]
	LEP	3.59 (0.63)					
Provide specific information about child	EP	3.7 (0.55)	−0.14 [−0.20, −0.10]	−7.91	1531	4.86 × 10^−16^	0.26 [0.22, 0.30]
	LEP	3.56 (0.69)					
Show sensitivity to family’s values and customs	EP	3.72 (0.55)	−0.22 [−0.27, −0.17]	−10.98	1515	2.20 × 10^−16^	0.39 [0.32, 0.46]
	LEP	3.5 (0.74)					
Help respondent feel like a partner in child’s care	EP	3.69 (0.58)	−0.15 [−0.20, −0.10]	−8.04	1537	1.72 × 10^−15^	0.26 [0.19, 0.33]
	LEP	3.54 (0.71)					

Analyses aimed to find differences in mean scores of caregiver satisfaction.

*LEP*: limited English proficiency sample*; EP*: English proficiency sample; *M*: mean; *SD*: standard deviation; *M*: difference, difference in means; *t*: t-score; *df*: degrees of freedom; Sig: probability value; 99% CI: 99% confidence interval; *d*: Cohen’s *d*.

## Discussion

This study analyzed national data for children and their families to compare satisfaction of LEP to EP families regarding child primary care services. It also identified areas of satisfaction with the greatest disparities between groups. Linguistic diversity—and thereby, limited English proficiency—is rapidly increasing in the United States. Outcomes associated with LEP status have been somewhat well-documented for adults in hospital care; however, little research has investigated how LEP impacts pediatric and non-urgent medicine, especially related to how patients perceive their care experience. LEP families felt less often, on average, that their healthcare provider spent enough time with their child, listened to their family, provided specific information, demonstrated sensitivity to family values and customs, and helped the family feel like a partner in their child’s care. The largest disparities, identified through descriptive statistics and effect size, were time spent with child and sensitivity towards family customs and values.

This study’s findings are consistent with existing studies on how LEP status impacts general patient satisfaction (Al Shamsi et al., 2020; Diamond et al., 2019; Yeheskel and Rawal, 2019). A systematic review by Diamond et al. (2019) which reviewed studies from diverse backgrounds, age groups, and clinical settings found that in most studies, EP patients were more likely to have a better patient experience, agreed with medical advice more frequently, felt more satisfied with their care, and left with heightened medical understanding. These conclusions are supported by findings that providers, regardless of service location or level of experience, identified language as a significant barrier to quality (Ali and Watson, 2017; Chalabian and Dunnington, 1997) and even admitted to avoiding communication with families experiencing LEP (Burbano O’Leary et al., 2003). Taken together, this may explain why LEP families felt less frequently that time spent with their child was satisfactory.

Reports have also observed that culture and language may be associated, and thus perceived decreases in service quality around communication will likely decrease for culture as well (Chen et al., 2009; Yeheskel and Rawal, 2019). The intersections between culture and language frame the differences in frequency at which LEP and EP caregivers felt their family’s values and customs were respected.

There is evidence to suggest that caregivers with LEP struggle more to recognize or express concerns regarding specific details of their child’s care, which could lead to medical oversights or adverse events (Khan et al., 2020). Although all clinical settings are required to have strategies to reduce language-relate barriers, most providers lack guidelines, training, and funding to implement an effective solution (Jacobs et al., 2018). These challenges are in line with LEP caregivers reporting reduced averages in feeling like healthcare providers offered specific information or listened to them.

LEP-status patients are less likely to adhere to medical advice at home (Dilworth et al., 2009) due to a lack of understanding of what an intervention comprises or not receiving enough time to process and ask questions (Jang et al., 2017; Zurca et al., 2017). With confusion regarding how to follow through with care, LEP caregivers are less likely to feel like a partner in their child’s health. While the above evidence assists in contextualizing the paper’s findings, not all sources provided refer to pediatric populations in primary care settings. As previously mentioned, most American data regarding language discordance in infant, child, and adolescent care only exists for hospitals or in relation to a specific medical condition.

The implications of this study are broad. Indeed, efforts aiming to improve pediatric primary healthcare should recognize families with LEP as an at-risk group and language concordance as a priority. This is especially true for Spanish-sparking families, who consist of 41 million people or 13% of the nation’s population, making Spanish the United States’ second most dominant language. Policymakers should prioritize disproportionate burdens LEP caregivers face with regards to provider time spent with child, and sensitivity towards customs and values. Means of reducing language-related barriers should be accurate, timely, and reasonably accessible in all clinical settings. However, further research is needed to understand ideal conditions under which families with LEP will thrive (Gany et al., 2007).

Interpretation, the most widely discussed method of aiding LEP populations, aims to increase specificity of information shared with caregivers and how well a provider listens. There have been mixed results in studies comparing patient experiences with an interpreter and without one (Flores, 2005). When using ad hoc interpreters, which is most common when patients require translation, in a pediatric clinic, medical errors were significantly more likely to be clinically relevant than when using professional interpreters (Flores et al., 2003). However, when introducing trained services, while some studies found that interpretation improved patient understanding and comfort (Jang et al., 2017; Kline et al., 1980), others reported that patients found their interpreters rude and did not feel an improvement in their experience (Brooks et al., 2000).

Healthcare providers also did not feel secure in linguistically discordant interactions using interpreters. They cited their confidence in several aspects of satisfaction, from patient eagerness to return to the physician’s own ability to communicate, as lower than interactions in English (Ali and Watson, 2017; Kline et al., 1980). There have also been mixed results with regards to patient satisfaction and different interpretation media (Gany et al., 2007; Schlange et al., 2022). Taken together, these papers point to a fundamental lack of standardization and funding in medical interpretive training. The research also demonstrates a dearth of data specific to linguistic minorities and a need to train physicians and healthcare providers to work with interpreters.

Another solution, albeit more complex, has also been discussed: satellite clinics that cater to demands of a specific group. For instance, satellite clinics can be tailored to immigrant families or individuals who speak a certain language. These clinics would be responsible for (1) allocating linguistic concordant providers (or interpretation) and appropriate time for each child and (2) training staff to be culturally aware, ultimately leading to (3) caregivers feeling like partners in their child’s care. Although there are few North American satellite clinics, those that exist are long-standing and report that they are providing a relevant service (Fowler, 1998). They promote better health outcomes, social integration and support, health education, and cultural familiarity recognized by both provider and patients (Minkler, 1983). Despite such benefits of satellite clinics, they are difficult to support financially and may not be feasible in rural areas or for families who speak less common non-English languages.

### Study limitations

The present study is not without limitations. Based on existing evidence from Ashman et al. (2019), nearly every American child has a primary care provider and visits are preventative in nature. Therefore, it was assumed that most cases were primary and preventative for purposes of interpretation. However, as this study’s sample is drawn from the general population and not a clinical sample, questions related to satisfaction did not specify contexts under which caregivers and their child met with a physician or other healthcare provider. It cannot be said for certain that all data included in this study were from non-hospital visits. Understanding the exact context under which care was received and how LEP status impacts specific settings are important aspects of prioritizing interventions. Furthermore, it was not specified whether caregivers had some form of interpretation available to them from the healthcare center or arranged by their family (ad hoc or trained). Specific household language, outside of Spanish, was also not documented. Primary household language, access to interpretation, and type of interpretation all make a difference in care and satisfaction; these variables should be noted and controlled for during analyses. Finally, this study lacked qualitative evidence that could supplement to contextualize family experiences navigating LEP.

### Implications for practice

Diversity in language, culture, and geographical distribution implies that a combination of strategies and increased evidence are required (Bowen, 2001). Conflicting results may hint at linguistic discordant outcomes in healthcare being intertwined with other demographic characteristics that have yet to be concurrently investigated such as race, socioeconomic status, religion, or education level.

It is likely there is no straightforward solution to navigating LEP in pediatric primary care, but principles of evidence-based practice (EBP) can be followed for improved outcomes. EBP integrates the perspectives of clinicians, LEP patients, and high-quality evidence to design policies, tailor interventions, and evaluate decisions (Sackett et al., 1996). For instance, an implementation team for interpretive services at a healthcare center may wish to conduct qualitative interviews with staff members and patients, perform observational studies to quantitatively measure patient experience, and modify the intervention based on these results. Based on the limitations of this study, future studies must accurately document reasons for care, levels of linguistic discordance and existing strategies to mitigate, and variables for patient outcomes and satisfaction.

Schools and other social services working with LEP families should consider prioritizing access to health literacy, in addition to everyday conversational skills. Health literacy’s purpose is to develop a precise vocabulary surrounding health issues, reduce risky behaviors, and promote healthy decision-making (Auld et al., 2020; Liu et al., 2020). Education can be formal or informal. With this literacy, families with LEP can better communicate their concerns and advocate for their needs in healthcare encounters (Liu et al., 2020).

If designed correctly, LEP interventions have been shown to not only improve patient satisfaction and outcomes but also come at little net cost to the healthcare system (Brandl et al., 2020). The end goal is that young patients and their caregivers feel more satisfied with services they receive. Satisfaction builds trusting, provider–patient relationships that encourage families to engage in preventative actions, ask questions and share details, and return to the service, ultimately improving long-term health for LEP-status families and their children (Flores, 2005; Skoog et al., 2018).

## Conclusion

The ability to communicate efficiently, compassionately, and accurately in pediatric healthcare is essential. This study analyzed national data from the United States to compare satisfaction of caregivers with LEP to caregivers with EP regarding their experience receiving primary care for a child. Most respondents were reasonably satisfied with their child’s care. However, LEP caregivers felt, on average, less satisfied with the amount of time a healthcare provider spent with their child, specificity of information provided, and sensitivity to family values and customs. They also felt less frequently that healthcare providers listened to their family and helped their family feel like a partner in their child’s care. This study highlights a need to improve experiences of LEP families in pediatric care to benefit overall child health outcomes. Future work with LEP families should follow the principles of EBP, which include the following: developing nuanced measures of LEP healthcare (such as the context of care and availability of interpretation), investigating the differential impact of LEP on various subgroups, considering clinician and patient perspectives, tailoring LEP-related interventions, and assessing the quality of interventions.

## Supplemental Material

Supplemental Material - Caregivers with limited English proficiency: Satisfaction with primary pediatric healthcare Supplemental Material for Caregivers with limited English proficiency: Satisfaction with primary pediatric healthcare by Linda Thanh Duong in Journal of Child Health Care.

## Data Availability

The 2019 NSCH data is publicly available: Child and Adolescent Health Measurement Initiative. 2019 National Survey of Children’s Health (NSCH). Data Resource Center for Child and Adolescent Health supported by Cooperative Agreement U59MC27866 from the U.S. Department of Health and Human Services, Health Resources and Services Administration (HRSA), and Maternal and Child Health Bureau (MCHB). Retrieved [08/20/2022] from https://www.census.gov/programs-surveys/nsch/data/datasets.2019.html.

## References

[bibr1-13674935241252479] Al Shamsi H Almutairi AG Al Mashrafi S , et al. (2020) Implications of language barriers for healthcare: a systematic review. Oman Medical Journal 35(2): e122, Available at: DOI: 10.5001/omj.2020.40. 32411417 PMC7201401

[bibr2-13674935241252479] Ali PA Watson R (2018) Language barriers and their impact on provision of care to patients with limited English proficiency: nurses’ perspectives. Journal of Clinical Nursing 27(5–6): e1152–e1160, Available at: DOI: 10.1111/jocn.14204. 29193568

[bibr3-13674935241252479] Ashman JJ Rui P Titilayo O (2019) Characteristics of Office-Based Physician Visits, 2016. Atlanta, GA: CDC. Available at: https://www.cdc.gov/nchs/products/databriefs/db331.htm (Accessed 30 July 2022). 30707670

[bibr4-13674935241252479] Auld ME Allen MP Hampton C , et al. (2020) Health literacy and health education in schools: collaboration for action. National Academy of Medicine Perspectives. DOI: 10.31478/202007b. PMC8916818 35291735

[bibr5-13674935241252479] Bartz AE (1999) Basic Statistical Concepts. 4th edition. Upper Saddle River, NJ: Merrill.

[bibr6-13674935241252479] Bowen S (2003) Language Barriers in Access to Health Care. Health Canada: Canada. Available at: https://www.canada.ca/en/health-canada/services/health-care-system/reports-publications/health-care-accessibility/language-barriers.html (Accessed 11 November 2022).

[bibr8-13674935241252479] Brandl EJ Schreiter S Schouler-Ocak M (2020) Are trained medical interpreters worth the cost? a review of the current literature on cost and cost-effectiveness. Journal of Immigrant and Minority Health 22(1): 175–181, Available at: DOI: 10.1007/s10903-019-00915-4. 31256314

[bibr9-13674935241252479] Brooks N Magee P Bhatti G , et al. (2000) ‘Asian patients’ perspective on the communication facilities provided in a large inner city hospital. Journal of Clinical Nursing 9(5): 706–712, Available at: DOI: 10.1046/j.1365-2702.2000.00397.x.

[bibr10-13674935241252479] Burbano O’Leary SC Federico S Hampers LC (2003) The truth about language barriers: one residency program’s experience. Pediatrics 111(5): e569–e573, Available at: DOI: 10.1542/peds.111.5.e569. 12728111

[bibr11-13674935241252479] CAMHI (2009) ‘Measuring Medical Home for Children and Youth: Methods and Findings From the National Survey of Children With Special Health Care Needs and the National Survey of Children’s Health. Boca Raton, FL: CAMHI. Available at: https://www.childhealthdata.org/docs/medical-home/mhmanual_withappendices-updated-12-7-10-pdf (Accessed 11 November 2022).

[bibr12-13674935241252479] Chalabian J Dunnington G (1997) Impact of language barrier on quality of patient care, resident stress, and teaching. Teaching and Learning in Medicine 9(2): 84–90, Available at: DOI: 10.1080/10401339709539820.

[bibr13-13674935241252479] Chen AW Kazanjian A Wong H (2009) Why do Chinese Canadians not consult mental health services: health status, language or culture? Transcultural Psychiatry 46(4): 623–641, Available at: DOI: 10.1177/1363461509351374. 20028680

[bibr14-13674935241252479] Cohen J (1988) Statistical Power Analysis for the Behavioral Sciences. 2 edition. London: Routledge. Available at: DOI: 10.4324/9780203771587.

[bibr15-13674935241252479] Coughlin KW (2018) Medical Decision-Making in Paediatrics: Infancy to Adolescence. Ottawa, ON: Canadian paediatric society. Available at: https://cps.ca/en/documents//position//medical-decision-making-in-paediatrics-infancy-to-adolescence/ (Accessed 11 November 2022). 10.1093/pch/pxx127 PMC5905503 30653623

[bibr16-13674935241252479] Diamond L Izquierdo K Canfield D , et al. (2019) A systematic review of the impact of patient–physician non-English language concordance on quality of care and outcomes. Journal of General Internal Medicine 34(8): 1591–1606, Available at: DOI: 10.1007/s11606-019-04847-5. 31147980 PMC6667611

[bibr17-13674935241252479] Dilworth TJ Mott D Young H (2009) Pharmacists’ communication with Spanish-speaking patients: a review of the literature to establish an agenda for future research. Research in Social and Administrative Pharmacy: RSAP 5(2): 108–120, Available at: DOI: 10.1016/j.sapharm.2008.05.005. 19524859 PMC2875142

[bibr18-13674935241252479] Dobson R (2007) US hospital patients with poor English have more serious adverse events than proficient speakers. BMJ 334(7589): 335, Available at: DOI: 10.1136/bmj.39125.457535.94.

[bibr19-13674935241252479] Donaldson MS (ed). (1996) Primary Care: America’s Health in a New Era. Washington, DC: National Academies Press (US). Available at: https://www.ncbi.nlm.nih.gov/books/NBK232643/ (Accessed 11 November 2022). 25121221

[bibr20-13674935241252479] Flores G (2005) The impact of medical interpreter services on the quality of health care: a systematic review. Medical Care Research and Review: MCRR 62(3): 255–299, Available at: DOI: 10.1177/1077558705275416. 15894705

[bibr21-13674935241252479] Flores G Laws MB Mayo SJ , et al. (2003) Errors in medical interpretation and their potential clinical consequences in pediatric encounters. Pediatrics 111(1): 6–14, Available at: DOI: 10.1542/peds.111.1.6. 12509547

[bibr22-13674935241252479] Fowler N (1998) Providing Primary Health Care to Immigrants and Refugees: The North Hamilton Experience. Ottawa, ON: CMAJ. Available at: https://www.nlc-bnc.ca/eppp-archive/100/201/300/cdn_medical_association/cmaj/vol-159/issue-4/0388.htm. PMC1229607 9732723

[bibr23-13674935241252479] Gany F Leng J Shapiro E , et al. (2007) Patient satisfaction with different interpreting methods: a randomized controlled trial. Journal of General Internal Medicine 22(S2): 312–318, Available at: DOI: 10.1007/s11606-007-0360-8. 17957417 PMC2078551

[bibr24-13674935241252479] Gill L White L (2009) A critical review of patient satisfaction. Leadership in Health Services 22(1): 8–19, Available at: DOI: 10.1108/17511870910927994.

[bibr25-13674935241252479] Goldman B (2017) Let’s Talk: Why Canadians Need Health Care in Their Own Language | CBC Radio. Ottawa, ON: CBC. Available at: https://www.cbc.ca/radio/whitecoat/blog/let-s-talk-why-canadians-need-health-care-in-their-own-language-1.4356139 (Accessed 30 July 2022).

[bibr26-13674935241252479] Hall DE Prochazka AV Fink AS (2012) Informed consent for clinical treatment: Figure 1. Canadian Medical Association Journal 184(5): 533–540, Available at: DOI: 10.1503/cmaj.112120. 22392947 PMC3307558

[bibr27-13674935241252479] Hasnain-Wynia R Yonek J Pierce D et al. (2006) Hospital Language Serices for Patients With Limited English Proficiency: Results From a National Survey. Chicago, IL: Health Research & Educational Trust and National Health Law Program, Available at: https://www.issuelab.org/resources/9722/9722.pdf.

[bibr28-13674935241252479] Herzberg EM Barrero-Castillero A Matute JD (2022) The healing power of language: caring for patients with limited English proficiency and COVID-19. Pediatric Research 91(3): 526–528, Available at: DOI: 10.1038/s41390-021-01487-6. 33790416 PMC8010487

[bibr29-13674935241252479] Jacobs B Ryan AM Henrichs KS , et al. (2018) Medical interpreters in outpatient practice. The Annals of Family Medicine 16(1): 70–76, Available at: DOI: 10.1370/afm.2154. 29311179 PMC5758324

[bibr30-13674935241252479] Jang M Plocienniczak MJ Mehrazarin K , et al. (2018) Evaluating the impact of translated written discharge instructions for patients with limited English language proficiency. International Journal of Paediatric Otorhinolaryngology 111: 75–79, Available at: DOI: 10.1016/j.ijporl.2018.05.031. 29958619

[bibr31-13674935241252479] John-Baptiste A Naglie G Tomlinson G , et al. (2004) The effect of English language proficiency on length of stay and in-hospital mortality. Journal of General Internal Medicine 19(3): 221–228, Available at: DOI: 10.1111/j.1525-1497.2004.21205.x. 15009776 PMC1492154

[bibr32-13674935241252479] Karliner LS Pérez-Stable EJ Gregorich SE (2017) Convenient access to professional interpreters in the hospital decreases readmission rates and estimated hospital expenditures for patients with limited English proficiency. Medical Care 55(3): 199–206, Available at: DOI: 10.1097/MLR.0000000000000643. 27579909 PMC5309198

[bibr33-13674935241252479] Khan A Yin HS Brach C Patient and Family Centered I-PASS Health Literacy Subcommittee , et al. (2020) Association between parent comfort with English and adverse events among hospitalized children. JAMA Paediatrics 174(12): e203215, Available at: DOI: 10.1001/jamapediatrics.2020.3215. PMC7573792 33074313

[bibr43-13674935241252479] Kline F Acosta FX Austin W , et al. (1980) The misunderstood Spanish-speaking patient. The American Journal of Psychiatry 137(12): 1530–1533. DOI: 10.1176/ajp.137.12.1530. 7435708

[bibr34-13674935241252479] Levine DM Linder JA Landon BE (2020) Characteristics of Americans with primary care and changes over time, 2002-2015. JAMA Internal Medicine 180(3): 463–466, Available at: DOI: 10.1001/jamainternmed.2019.6282. 31841583 PMC6990950

[bibr35-13674935241252479] Liu C Wang D Liu C , et al. (2020) What is the meaning of health literacy? a systematic review and qualitative synthesis. Family Medicine and Community Health 8(2): e000351, Available at: DOI: 10.1136/fmch-2020-000351. 32414834 PMC7239702

[bibr36-13674935241252479] Minkler DH (1983) The role of a community-based satellite clinic in the perinatal care of non-English-speaking immigrants. Western Journal of Medicine 139(6): 905–909. 6666108 PMC1011023

[bibr37-13674935241252479] Organisation for Economic Co-Operation and Development (2017) International Migration outlook 2017. Paris: OECD, Available at: DOI: 10.1787/migr_outlook-2017-en.

[bibr38-13674935241252479] Phillips RL (2005) Primary care in the United States: problems and possibilities. BMJ 331(7529): 1400–1402. 16339255 10.1136/bmj.331.7529.1400 PMC1309658

[bibr39-13674935241252479] Proctor K Wilson-Frederick SM Haffer SC (2018) The limited English proficient population: describing Medicare, Medicaid, and dual beneficiaries. Health Equity 2(1): 82–89, Available at: DOI: 10.1089/heq.2017.0036. 30283853 PMC6071899

[bibr40-13674935241252479] Sackett DL Rosenberg WM Gray JA , et al. (1996) Evidence based medicine: what it is and what it isn’t. BMJ 312(7023): 71–72, Available at: DOI: 10.1136/bmj.312.7023.71. 8555924 PMC2349778

[bibr41-13674935241252479] Schlange SA Palmer-Wackerly AL Chaidez V (2022) A narrative review of medical interpretation services and their effect on the quality of health care. Southern Medical Journal 115(5): 317–321, Available at: DOI: 10.14423/SMJ.0000000000001392. 35504613

[bibr42-13674935241252479] Skoog M Berggren V Hallström IK (2019) “Happy that someone cared”—non-native-speaking immigrant mothers’ experiences of participating in screening for postpartum depression in the Swedish child health services. Journal of Child Health Care: For Professionals Working with Children in the Hospital and Community 23(1): 118–130, Available at: DOI: 10.1177/1367493518778387. 29804463 PMC7324125

[bibr44-13674935241252479] Yeheskel A Rawal S (2019) Exploring the “patient experience” of individuals with limited English proficiency: a scoping review. Journal of Immigrant and Minority Health 21(4): 853–878, Available at: DOI: 10.1007/s10903-018-0816-4. 30203377

[bibr45-13674935241252479] Zurca AD Fisher KR Flor RJ , et al. (2017) Communication with limited English-proficient families in the PICU. Hospital Pediatrics 7(1): 9–15, Available at: DOI: 10.1542/hpeds.2016-0071. 27979992 PMC5740871

