# Supplementary material for: Caregivers with limited English proficiency: Satisfaction with primary pediatric healthcare
Source: J Child Health Care. 2024 May 8;29(4):782–95. doi: 10.1177/13674935241252479 (PMC12662825; doi:10.1177/13674935241252479)
Supplement: Supplemental Material - Caregivers with limited English proficiency: Satisfaction with primary pediatric healthcare [file sj-pdf-1-chc-10.1177_13674935241252479.pdf]

APPENDIX

Supplementary Table 1. *Results of Mann-Whitney U test comparing caregiver satisfaction with provider between families with limited English proficiency and with English proficiency*

	W	**Sig (2-tailed)**	***r* [99% CI]**
**Spend enough time with child**	14256838	2.20E-16	0.17 [0.13, 0.20]
**Listen to respondent**	15639730	7.61E-09	0.08 [0.04, 0.11]
**Provide specific information about child**	15100806	2.20E-16	0.11 [0.07, 0.15]
**Show sensitivity to family's values and customs**	14305512	2.20E-16	0.17 [0.13, 0.20]
**Help respondent feel like partner in child's care**	14996603	2.20E-16	0.11 [0.08, 0.15]

W, Mann-Whitney statistic; Sig, probability value; *r*, rank-biserial correlation; 99% CI, 99% confidence interval.

Analyses aimed to find differences in the sum of ranks of caregiver satisfaction.
